# A rare cause of right ventricle out flow tract obstruction: Anterior mediastinal teratoma

**DOI:** 10.1016/j.amsu.2021.01.043

**Published:** 2021-01-22

**Authors:** Ikram ul Haq Chaudhry, Ahsan Cheema, Chaudhry Aqeel, Ahmed A. Alshaer, Fahad G. Alradei, Mohiudin G. Ali

**Affiliations:** Department of Thoracic Surgery King Fahad Specialist Hospital Dammam Saudi Arabia, Saudi Arabia

**Keywords:** Mediastinal mass, Right ventricle, Pulmonary artery, Surgery

## Abstract

A 36 years old female presented with six months history of shortness of breath, fatigue, and tiredness. Her chest X-Ray showed a left mediastinal mass. A computed tomographic scan (CT)of the chest revealed a left mediastinal mass, exhibiting typical teratoma features. An echocardiogram showed compression of the main pulmonary artery causing right ventricular out flow tract obstruction. The tumor was resected through a left thoracotomy, and the patient improved dramatically after surgery. She was discharged home for further follow-up.

## Highlights

• A 36 years old female presented with six months' history of.
• Shortness of breath, fatigue, and tiredness.
• A computed tomographic scan (CT)of the chest revealed a.
• Left mediastinal mass, exhibiting typical teratoma features.
• An echocardiogram showed compression of the main pulmonary artery.
• Complete surgical resection relieved right ventricular outflow tract obstruction.

## 1 Introduction

**Fig. 1:**
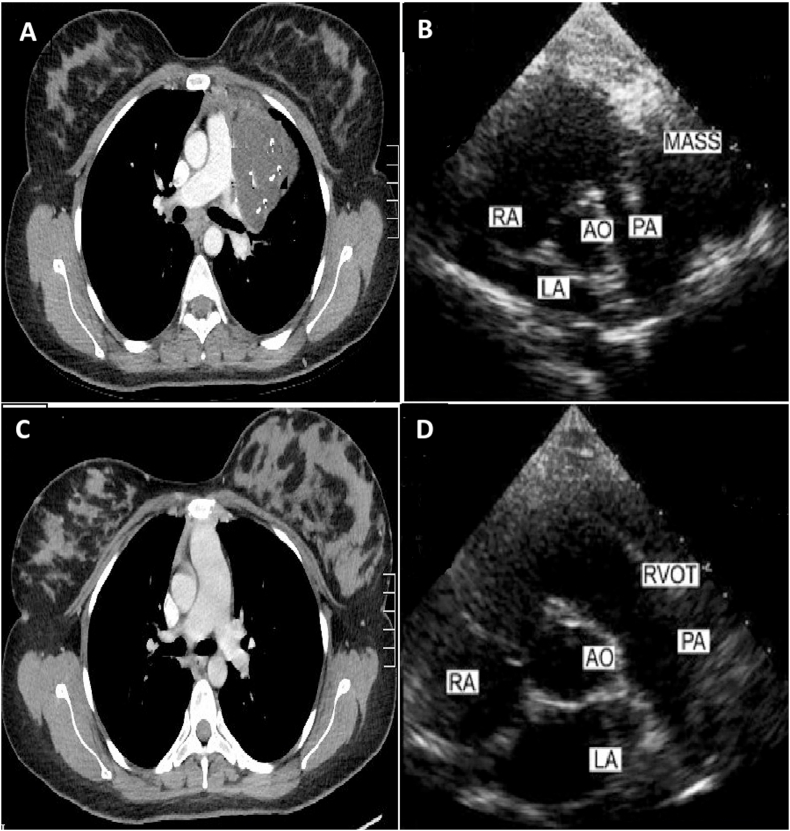
(A) CT scan of thorax showing anteruior mediastinal mass compressing the main pulmonary artery.(B) Preoperative ECHO demonstrate copreson of right venticular outflow tract.(C)Post operative CT can of thrax showing full expansion of pulmonary artery.(D)Post operative ECHO demonstrating release of right ventricular outflow tract obstruction.

**Fig. 2:**
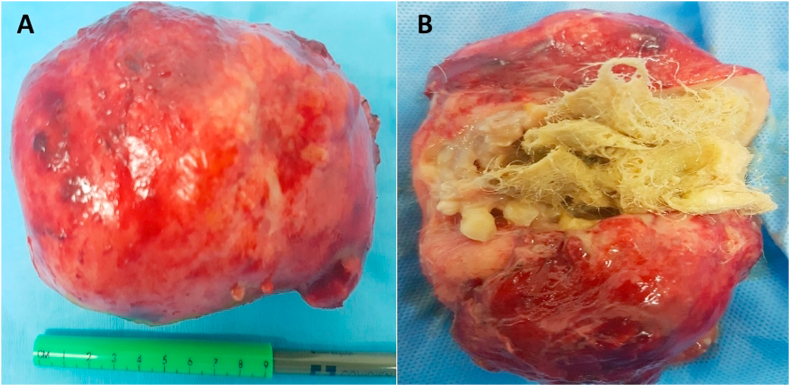
(A) Gross specimen of anterior medistinal mass after removal.(B) Mass opened to demonstrate hair, teeth, bone exhibiting typical features of teratoma.

Liebert et al. described the omental dermoid cyst(teratoma) in 1734. Maier et al. described the first case of anterior mediastinal teratoma. In 1948 [1,2]. Teratoma commonly occurs in the young population, and the incidence is approximately the same in both genders. (see Fig. 1, Fig. 2)

Teratoma is a multipotent tumor of germ cell origin. The constituent tissues may exhibit their normal physiological function. Cases reported to date include sebaceous glands, hair, teeth, bone and tissue secretion of chorionic gonadotrophin, thyroid hormone, a pancreatic enzyme, and insulin [3]. Thoracic teratoma can occur in any location in the mediastinum, rare they are found intrapulmonary. This case has been reported in line with SCARE criteria [4].

### 1.1 Case presentation

A 41-year nonsmoker female presented with a six-month history of shortness of breath on exertion, no history of fever, night sweat, or weight loss. On clinical examination, the chest was symmetrical, with no palpable lymphadenopathy, there was diminished air entry in the upper left chest. A chest X-ray showed a mass in the left upper chest. Necessary blood investigations were within the normal range. Alfafeto protein and beta-human chronotropic HCG hormone levels were normal. CT scan of the chest revealed a large heterogeneous mass in the left upper zone of the chest. Transthoracic echocardiography (ECHO) revealed compression of the main pulmonary artery. Complete resection of the mass was achieved through left posterolateral thoracotomy after meticulous dissection of adhesions with pulmonary artery and pericardium. One chest drain 32F was placed in the pleural cavity, and the chest was closed in layers. The patient was extubated on the table. The histology report showed features of ‘‘Mature Teratoma’ ‘Her postoperative recovery was uneventful, and she was discharged for further follow up in outpatient.

## 2 Discussion

Teratoma is an uncommon germ cell tumor that arises in gonads. The mediastinum is the second most common place to harbor teratoma after the gonads. In adults, 10–15% of anterior mediastinal masses are germ cell tumors, and among them, teratoma is the most common [5].

These tumors originate from totipotent cells due to their abnormal migration during embryonic development. The undifferentiated cells arising from the third pharyngeal pouch and foregut migrate along the primordial thymus to rest in the mediastinum. These constituent tissues are latter on differentiating into cells of three embryonic germ layers, skin sebaceous &sweat glands and teeth (Ectoderm), Muscles, fat, cartilage, and bone (Mesoderm), Respiratory and intestinal epithelium (Endoderm) [6]. Intrapulmonary teratomas are formed by the migration of pluripotent cells along the developing lung bud derived from the ventral foregut. Benign teratomas are mostly asymptomatic and are usually diagnosed as an incidental finding on chest x-ray [7]. Mediastinal mature teratoma is asymptomatic in 50–60% of patients. On the other hand, 36–41 %of patients can present with symptoms of perforation such as fever, chest pain, hemoptysis, and spiting tumor contents [8,9].

As mediastinal teratoma grows slowly, it becomes symptomatic only when it causes the mass effect on the surrounding structures, it can rupture into the tracheobronchial tree leading to chest pain hemoptysis or trichophytisis [10]. Two mechanisms of perforation have been postulated. It may be due to inflammation, infection, ischemia, and necrosis caused by the sebaceous part of the tumor, or it may be due to autolysis caused by the digestive enzyme released by the salivary and pancreatic component of the tumor [11].

Intrapulmonary teratomas are most commonly located in the upper lobe (65%) and may have direct communication with the airway. Such patients can present with dry or productive cough, fever, chest pain, and weight loss [12,13]. No infective inflammation around mediastinal teratoma has been reported in the medical literature. Cooper and Ankeny reported teratoma perforation into the pericardium. Similarly, Rosenbluth, Steinberg & Dotter [14] described a case of pericardial abscess due to perforation of mediastinal teratoma [15,16]. Rupture into the pleural cavity can lead to pleural effusion and, later on, abscess formation. And bronchopleural fistula [17].

Rarely mediastinal teratoma can present with pulmonary artery compression mimicking valvular heart disease. To date, only fifteen cases of pulmonary artery stenosis caused by the mass effect of anterior mediastinal teratoma have been documented in the medical literature [17,18].

In such cases, the only finding is systolic murmur masquerading as valvular heart disease to the clinician. Therefore, ECHO is essential to rule out cardiac pathology. The pulmonary artery compression leading to right ventricle outflow obstruction due to B cell lymphoma, Hodgkin disease, lung carcinoma, pericardial sarcoma, thymoma, and chondrosarcoma has been reported in the medical literature. [19,20]^,^

A chest x-ray and CT scan of the thorax are the best diagnostic tools. MRI scans can be useful to evaluate the relation with vascular structures. Histopathology has a significant role as the prognosis depends upon the nature of the tumor as the malignant teratoma has a poor prognosis.

Complete surgical resection is the standard of treatment because rarely benign teratoma can go into malignant transformation. Chemotherapy may provide some survival benefit in malignant cases [21].

In conclusion, we report a rare case of benign mediastinal teratoma, causing right ventricular outflow tract obstruction due to mass effect on the main pulmonary trunk. After total excision, the patient recovered completely, and a follow-up CT scan showed no recurrence, and the patient had a good quality of life.

## Registration of research study

Not applicable.

## Declaration of competing interest

No conflict of interest and there was no funding or financial assistance in this case.
